# Individual selection of augmentative and alternative communication for people with mental disabilities

**DOI:** 10.1192/j.eurpsy.2025.762

**Published:** 2025-08-26

**Authors:** A. L. Bitova, A. A. Portnova, O. V. Karanevskaya, M. E. Sisneva

**Affiliations:** 1 Center for Curative Pedagogics, Moscow, Russian Federation

## Abstract

**Introduction:**

More than 150,000 adults with mental disabilities live in social shelters in Russia and almost 50% of them do not use speech for communication and can’t communicate at all. Social institutions lack a system for training those in need of Augmentative and alternative communication (AAC).

**Objectives:**

Development and testing of an algorithm for selecting AAC method for adults with mental disorders and severe speech communication disorders.

**Methods:**

A questionnaire for people with mental disabilities who do not use speech for communication was developed and tested. It included questions accompanied by illustrations, photographs and graphic symbols. A survey was conducted among 50 people of both sexes, aged 17-58 living in social welfare institutions. 56% of them had a diagnosis of intellectual disability, 18% - Autism, 26% - Schizophrenia. An algorithm for preparing and including respondents in the study was developed, consisting of several components (Establishing primary contact, Determining the preferred means of communication, Determining the ability to understand and use oral speech, Determining the ability to understand and use gestures, images and symbols, written speech).

**Results:**

92% of people completed the full study. 4 respondents withdrew from the experiment due to their mental state (psychosis, passivity, profound intellectual disability, fatigue). Despite successfully completing the preparatory stage, 74% managed to obtain full results.

**Conclusions:**

The study made it possible to identify potential opportunities for using communication tools, analyze barriers in communication with others, and develop personal recommendations for using AAC, which will undoubtedly improve the quality of life of a person with mental disabilities.

**Disclosure of Interest:**

None Declared

